# Supplementary material for: DNA metabarcoding for high-throughput monitoring of estuarine macrobenthic communities
Source: Sci Rep. 2017 Nov 15;7:15618. doi: 10.1038/s41598-017-15823-6 (PMC5688171; doi:10.1038/s41598-017-15823-6)
Supplement: Supplementary file 1 — Supplementary Information [file 41598_2017_15823_MOESM1_ESM.pdf]

Supplementary Information

DNA metabarcoding for high-throughput monitoring of estuarine macrobenthic communities

Jorge Lobo[1,2,*], Shadi Shokralla[3], Maria Helena Costa[2], Mehrdad Hajibabaei[3], Filipe Oliveira Costa[1]

[1]CBMA – Centre of Molecular and Environmental Biology, University of Minho, Campus de Gualtar, 4710-057 Braga, Portugal

[2]MARE – Marine and Environmental Sciences Centre. New University of Lisbon. Campus de Caparica, 2829-516 Caparica, Portugal

[3]Centre for Biodiversity Genomics, Biodiversity Institute of Ontario and Department of Integrative Biology. University of Guelph. Guelph, ON N1G 2W1, Canada

[*] Corresponding author. j.loboarteaga@gmail.com

SI Figure

SI Tables

SI Figure

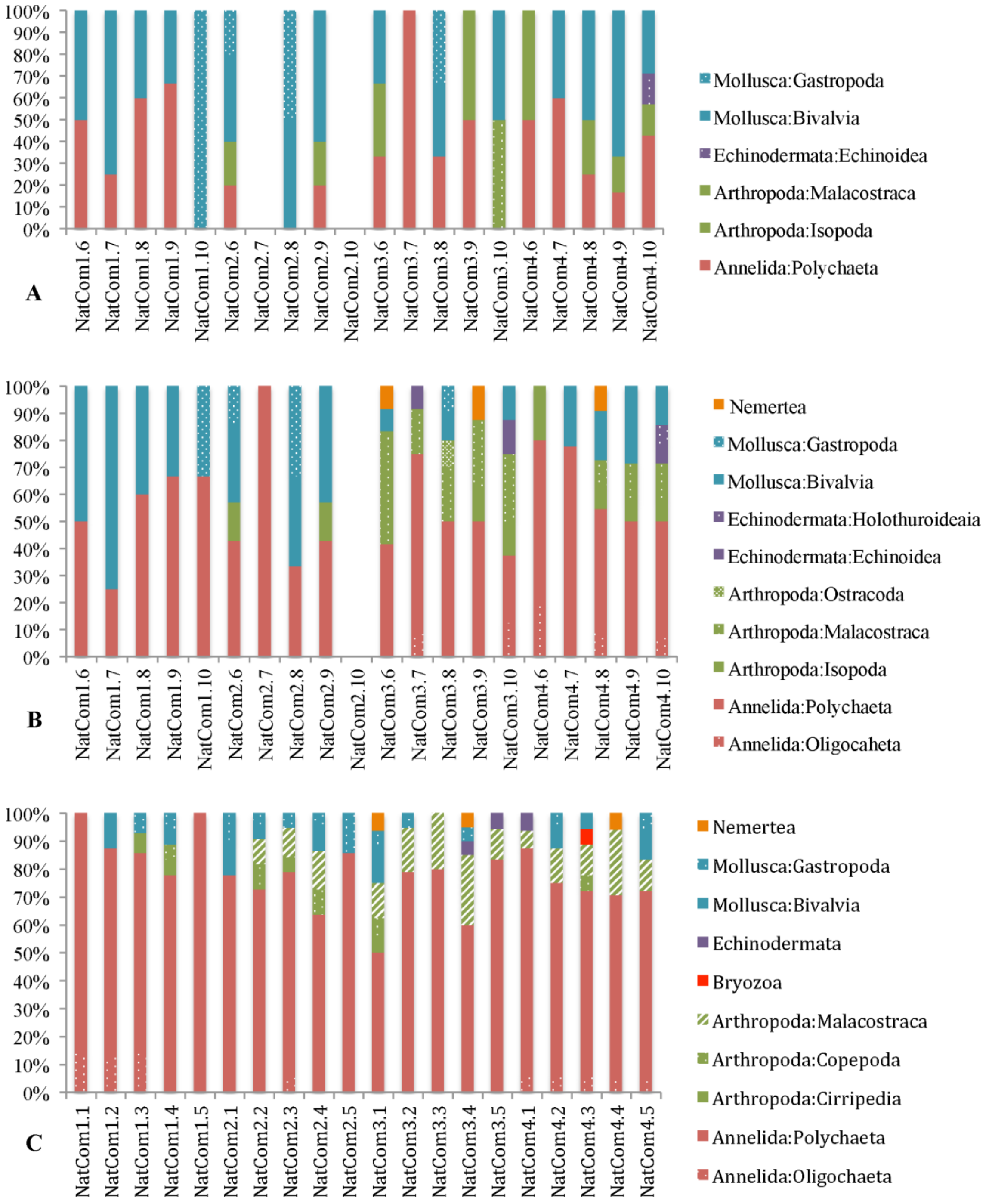

Figure S1: Species composition of all samples of NMC. A shows species composition considering only specimens morphologically identified to the species level. B considering specimens morphologically identified to a higher taxonomic level. C shows species composition recovered through HTS. No specimens were identified to the species level in NMC2.7. No specimens were collected in NMC2.10.

SI Tables

Table S1: Number of reads assigned to species in each NMC and primer pair.

	Primer pair B		Primer pair D	
	Our reference library of DNA barcodes	GenBank	Our reference library of DNA barcodes	GenBank
**NMC1.1**	160483	159067	12599	63949
**NMC1.2**	20607	623	7980	1207
**NMC1.3**	7592	37	7621	200
**NMC1.4**	374041	370148	20986	38273
**NMC1.5**	68404	56324	3474	44838
**NMC2.1**	2478	2386	3737	4856
**NMC2.2**	9916	9606	1721	2459
**NMC2.3**	150	704	544	17586
**NMC2.4**	153016	165386	110936	152695
**NMC2.5**	56	56	32	28
**NMC3.1**	126991	191736	53429	13195
**NMC3.2**	153152	148388	20397	16061
**NMC3.3**	160258	216194	158693	214413
**NMC3.4**	178432	207279	50386	50080
**NMC3.5**	100297	99112	48646	48256
**NMC4.1**	96110	151869	86172	31648
**NMC4.2**	159175	51999	134581	38645
**NMC4.3**	152206	112758	90353	69141
**NMC4.4**	223908	215030	46798	33450
**NMC4.5**	7601	24570	616	702

Table S2: Taxonomic classification of the species identified in each NMC through HTS (with the primer pairs B and D) and morphological identifications. Numbers indicate the number of reads (HTS) and number of specimens for each species.

						NMC1		
**Kingdom**	**Phylum**	**Class**	**Order**	**Family**	**Species**	**HTS B**	**HTS D**	**Morph.**
Animalia	Annelida	Polychaeta		Capitellidae	*Notomastus profondus* (Eisig, 1887)	-	0/553/0/0/0	-
				Maldanidae	*Axiothella constricta* (Claparède, 1869)	-	-	-
					Euclymene santandarensis (Rioja, 1917)	3/0/0/0/0	-	-
					Euclymene sp.	-	4/0/0/3/0	-
					Heteroclymene robusta Arwidsson, 1906	18/0/3/41/0	0/1058/0/0/0	-
					Leiochone leiopygos (Grube, 1860)	9/597/0/3/0	0/0/4/0/6	-
					Leiochone sp.	-	0/0/3/5/0	-
					Praxillella praetermissa (Malmgren, 1865)	0/0/22/23/0	27/0/54/7/0	-
				Orbiniidae	Orbiniidae ni	-	-	-
			Eunicida	Eunicidae	*Marphysa sanguinea* (Montagu, 1815)	-	4/0/0/0/8	0/1/0/2/0
					Marphysa sp.	-	-	-
				Onuphidae	*Diopatra aciculata* Knox & Cameron, 1971	-	0/0/0/3/0	-
					Diopatra marocensis Paxton, Fadlaoui & Lechapt, 1995	-	-	-
					Diopatra neapolitana Delle Chiaje, 1841	10/0/3/373857/30	0/0/0/38069/7	-
			Phyllodocida	Glyceridae	*Glycera alba* (O.F. Müller, 1776)	6/0/0/6/7	21/0/6/18/19	0/0/0/1/0
					Glycera sp.	-	14/0/6/0/0	-
					Glycera tridactyla Schmarda, 1861	-	-	-
				Nephtyidae	*Nephtys cirrosa* Ehlers, 1868	-	12433/0/0/5/1342	0/0/1/0/0
					Nephtys hombergii Savigny in Lamarck, 1818	160437/0/9/30/53510	63886/0/125/95/44291	1/0/0/0/0
				Nereididae	*Hediste diversicolor* (O.F. Müller, 1776)	0/24/0/0/0	0/104/0/0/0	0/0/1/0/0
				Phyllodocidae	*Phyllodoce groenlandica* Örsted, 1842	0/0/0/0/3832	0/0/0/0/507	-
				Polynoidae	*Paralepidonotus ampulliferus* (Grube, 1878)	-	0/10/0/0/0	-
			Sabellida	Oweniidae	*Owenia fusiformis* Delle Chiaje, 1844	-	0/0/0/3/0	-
			Terebellida	Cirratulidae	Cirratulidae ni	6/0/0/6/14864	3/0/0/3/1874	-
				Pectinariidae	*Lagis koreni* Malmgren, 1866	-	-	0/2/0/0/0
				Terebellidae	*Pista cristata* (Müller, 1776)	0/0/3/7/0	0/0/11/15/0	-
				Trichobranchinae	*Trichobranchus glacialis* Malmgren, 1866	0/19986/7552/0/0	0/6236/7543/10/0	-
		Clitellata	Haplotaxida	Tubificidae	*Chaetogaster diaphanus* (Gruithuisen, 1828)	0/4/0/0/0	4/0/0/0/0	-
					Nais communis Piguet, 1906	-	0/0/6/0/0	-

				Stylaria lacustris (Linnaeus, 1767)	-	3/0/5/0/0	-
		Lumbriculida	Lumbriculidae	*Lumbriculus variegatus* (Müller, 1774)	-	-	-
Arthropoda	Cirripedia	Sessilia	Balanidae	*Amphibalanus amphitrite* (Darwin, 1854)	0/0/4/0/0	0/0/0/16/0	-
	Copepoda	Calanoida	Acartiidae	*Paracartia grani* Sars G.O., 1904	0/0/0/3/0	-	-
			Centropagidae	*Centropages hamatus* (Lilljeborg, 1853)	-	-	-
			Temoridae	*Temora longicornis* (Müller O.F., 1785)	-	-	-
		Cyclopoida		Cyclopoida ni	-	-	-
		Poecilostomatoida	Mytilicolidae	*Mytilicola orientalis* Mori, 1935	-	-	-
	Malacostraca	Amphipoda	Ampeliscidae	*Ampelisca brevicornis* (Costa, 1853)	-	-	-
				Ampelisca sp.	-	-	-
			Corophiidae	*Corophium sp.*	-	-	-
		Isopoda	Anthuridae	*Cyathura carinata* (Krøyer, 1847)	-	-	-
			Sphaeromatidae	Sphaeromatidae ni	-	-	-
		Decapoda	Callianassidae	*Pestarella tyrrhena* (Petagna, 1792)	-	-	-
			Diogenidae	*Diogenes pugilator* (Roux, 1829)	-	-	-
			Inachidae	*Macropodia rostrata* (Linnaeus, 1761)	-	-	-
				Macropodia sp.	-	-	-
			Paguridae	Pylopaguropsis magnimanus	-	-	-
			Pilumnidae	*Pilumnus hirtellus* (Linnaeus, 1761)	-	-	-
				Pilumnus spinifer H. Milne Edwards, 1834	-	-	-
			Pinnotheridae	*Pinnotheres pisum* (Linnaeus, 1767)	-	-	
			Upogebiidae	*Upogebia deltaura* (Leach, 1815)	-	-	-
Bryozoa	Gymnolaemata	Cheilostomatida	Bugulidae	*Bugula neritina* (Linnaeus, 1758)	-	-	-
Echinodermata	Echinoidea	Spatangoida	Loveniidae	*Echinocardium cordatum* (Pennant, 1777)	-	-	-
Mollusca	Bivalvia	Anomalodesmata	Thraciidae	*Thracia phaseolina* (Lamarck, 1818)	-	-	-
		Myoida	Corbulidae	*Corbula gibba* (Olivi, 1792)	-	-	1/0/1/0/0
		Veneroida	Cardiidae	*Cerastoderma edule* (Linnaeus, 1758)	-	-	0/2/1/0/0
				Parvicardium exiguum (Gmelin, 1791)	-	-	-
				Parvicardium pinnulatum (Conrad, 1831)	-	-	-
			Donacidae	*Donax trunculus* Linnaeus, 1758	-	-	-
			Mactridae	*Spisula solida* (Linnaeus, 1758)	-	-	0/1/0/0/0
			Semelidae	*Abra alba* (W. Wood, 1802)	-	-	-
			Ungulinidae	*Diplodonta rotundata* (Montagu, 1803)	-	-	-
			Veneridae	*Chamelea striatula* (da Costa, 1778)	-	-	-

			[unassigned] Euheterodonta	Solenidae	*Solen marginatus* Pulteney, 1799	-	0/35/0/6/0	0/1/0/1/0
		Gastropoda	Littorinimorpha	Hydrobiidae	*Ecrobia ventrosa* (Montagu, 1803)	-	-	0/0/0/0/1
			Neogastropoda	Nassariidae	*Nassarius reticulatus* (Linnaeus, 1758)	0/0/0/76/0	0/0/0/51/0	-
	Nemertea	Anopla		Lineidae	*Cerebratulus longiceps* Coe, 1901	-	-	-
					Cerebratulus sp.	-	-	-
Plantae	Rhodophyta	Bangiophyceae	Bangiales	Bangiaceae	*Pyropia haitanensis* (T.J.Chang & B.F.Zheng) N.Kikuchi & M.Miyata, 2011	-	-	-
		Florideophyceae	Ceramiales	Ceramiaceae	*Ceramium secundatum* Lyngbye, 1819	-	-	-
Chromista	Ochrophyta	Phaeophyceae	Fucales	Durvillaeaceae	*Durvillaea sp.*	-	-	-
			Ectocarpales	Chordariaceae	*Leathesia marina* (Lyngbye) Decaisne, 1842	-	-	-
			Scytosiphonales	Scytosiphonaceae	*Petalonia fascia* (O.F.Müller) Kuntze, 1898	-	-	-
					Scytosiphon lomentaria (Lyngbye) Link, 1833	-	-	-

						**NMC2**		
**Kingdom**	**Phylum**	**Class**	**Order**	**Family**	**Species**	**HTS B**	**HTS D**	**Morph.**
Animalia	Annelida	Polychaeta		Capitellidae	*Notomastus profondus* (Eisig, 1887)	-	142/65/480/108	-
				Maldanidae	*Axiothella constricta* (Claparède, 1869)	0/4/0/0/0	-	-
					Euclymene santandarensis (Rioja, 1917)	0/33/10/0/0	0/0/0/3/0	-
					Euclymene sp.	0/17/3/0/0	-	-
					Heteroclymene robusta Arwidsson, 1906	0/0/51/0/0	0/0/4/4/0	-
					Leiochone leiopygos (Grube, 1860)	0/37/32/3/3	0/0/4/0/0	-
					Leiochone sp.	0/5/0/0/0	-	-
					Praxillella praetermissa (Malmgren, 1865)	15/120/31/0/8	0/0/0/9/0	-
				Orbiniidae	Orbiniidae ni	-	-	-
			Eunicida	Eunicidae	*Marphysa sanguinea* (Montagu, 1815)	0/52/11/3/0	-	-
					Marphysa sp.	-	-	-
				Onuphidae	*Diopatra aciculata* Knox & Cameron, 1971	-	-	-
					Diopatra marocensis Paxton, Fadlaoui & Lechapt, 1995	0/20/0/0/0	0/0/3/0/0	-
					Diopatra neapolitana Delle Chiaje, 1841	371/34/3/99/0	14/0/5/48/0	-
			Phyllodocida	Glyceridae	*Glycera alba* (O.F. Müller, 1776)	7/121/12/42234/10	4/7/0/32465/14	-
					Glycera sp.	6/123/13/43978/12	0/5/0/0/16	-
					Glycera tridactyla Schmarda, 1861	-	0/0/0/30/0	-
				Nephtyidae	*Nephtys cirrosa* Ehlers, 1868	-	0/0/0/48/0	-
					Nephtys hombergii Savigny in Lamarck, 1818	2042/9313/5/1066/35	547/361/0/1196/6	-

			Nereididae	*Hediste diversicolor* (O.F. Müller, 1776)	-	-	-
			Phyllodocidae	*Phyllodoce groenlandica* Örsted, 1842	-	-	-
			Polynoidae	*Paralepidonotus ampulliferus* (Grube, 1878)	-	-	-
		Sabellida	Oweniidae	*Owenia fusiformis* Delle Chiaje, 1844	0/3/0/11682/0	0/0/0/7085/0	-
		Terebellida	Cirratulidae	Cirratulidae ni	0/27/0/0/0	-	-
			Pectinariidae	*Lagis koreni* Malmgren, 1866	-	-	-
			Terebellidae	*Pista cristata* (Müller, 1776)	0/13/4/7/0	0/0/0/5/4	0/0/0/1/0
			Trichobranchinae	*Trichobranchus glacialis* Malmgren, 1866	5/0/0/0/0	0/0/4/0/0	1/0/0/0/0
	Clitellata	Haplotaxida	Tubificidae	*Chaetogaster diaphanus* (Gruithuisen, 1828)	-	-	-
				Nais communis Piguet, 1906	-	0/0/4/0/0	-
				Stylaria lacustris (Linnaeus, 1767)	-	-	-
		Lumbriculida	Lumbriculidae	*Lumbriculus variegatus* (Müller, 1774)	-	-	-
Arthropoda	Cirripedia	Sessilia	Balanidae	*Amphibalanus amphitrite* (Darwin, 1854)	0/0/567/0/0	0/017516/0/0	-
	Copepoda	Calanoida	Acartiidae	*Paracartia grani* Sars G.O., 1904	0/14//023/0	0/6/0/35/0	-
			Centropagidae	*Centropages hamatus* (Lilljeborg, 1853)	0/66/0/4/0	-	-
			Temoridae	*Temora longicornis* (Müller O.F., 1785)	-	-	-
		Cyclopoida		Cyclopoida ni	-	-	-
		Poecilostomatoida	Mytilicolidae	*Mytilicola orientalis* Mori, 1935	-	-	-
	Malacostraca	Amphipoda	Ampeliscidae	*Ampelisca brevicornis* (Costa, 1853)	-	-	-
				Ampelisca sp.	-	-	-
			Corophiidae	*Corophium sp.*	-	-	-
		Isopoda	Anthuridae	*Cyathura carinata* (Krøyer, 1847)	0/4/0/0/0	-	1/0/0/2/0
			Sphaeromatidae	Sphaeromatidae ni	-	-	-
		Decapoda	Callianassidae	*Pestarella tyrrhena* (Petagna, 1792)	-	-	-
			Diogenidae	*Diogenes pugilator* (Roux, 1829)	-	-	-
			Inachidae	*Macropodia rostrata* (Linnaeus, 1761)	-	-	-
				Macropodia sp.	-	-	-
			Paguridae	Pylopaguropsis magnimanus	0/4/0/0/0	0/0/45/0/0	-
			Pilumnidae	*Pilumnus hirtellus* (Linnaeus, 1761)	0/0/0/4/0	0/0/8/4/0	-
				Pilumnus spinifer H. Milne Edwards, 1834	-	0/0/0/3/0	-
			Pinnotheridae	*Pinnotheres pisum* (Linnaeus, 1767)	-	-	
			Upogebiidae	*Upogebia deltaura* (Leach, 1815)	0/0/0/4/0	-	-
Bryozoa	Gymnolaemata	Cheilostomatida	Bugulidae	*Bugula neritina* (Linnaeus, 1758)	-	-	-
Echinodermata	Echinoidea	Spatangoida	Loveniidae	*Echinocardium cordatum* (Pennant, 1777)	-	-	-

	Mollusca	Bivalvia	Anomalodesmata	Thraciidae	*Thracia phaseolina* (Lamarck, 1818)	-	-	-
			Myoida	Corbulidae	*Corbula gibba* (Olivi, 1792)	-	-	1/0/0/0/0
			Veneroida	Cardiidae	*Cerastoderma edule* (Linnaeus, 1758)	0/0/0/143/0	0/0/0/109/0	-
					Parvicardium exiguum (Gmelin, 1791)	-	-	0/0/1/0/0
					Parvicardium pinnulatum (Conrad, 1831)	-	-	0/0/0/1/0
				Donacidae	*Donax trunculus* Linnaeus, 1758	-	-	-
				Mactridae	*Spisula solida* (Linnaeus, 1758)	-	-	-
				Semelidae	*Abra alba* (W. Wood, 1802)	-	-	-
				Ungulinidae	*Diplodonta rotundata* (Montagu, 1803)	-	-	0/0/0/1/0
				Veneridae	*Chamelea striatula* (da Costa, 1778)	-	-	-
			[unassigned] Euheterodonta	Solenidae	*Solen marginatus* Pulteney, 1799	32/161/0/34/0	4288/2081/0/49/0	1/0/0/1/0
		Gastropoda	Littorinimorpha	Hydrobiidae	*Ecrobia ventrosa* (Montagu, 1803)	-	-	-
			Neogastropoda	Nassariidae	*Nassarius reticulatus* (Linnaeus, 1758)	7/82/6/110142/0	3/4/0/111689/9	1/0/1/0/0
	Nemertea	Anopla		Lineidae	*Cerebratulus longiceps* Coe, 1901	-	-	-
					Cerebratulus sp.	-	-	-
Plantae	Rhodophyta	Bangiophyceae	Bangiales	Bangiaceae	*Pyropia haitanensis* (T.J.Chang & B.F.Zheng) N.Kikuchi & M.Miyata, 2011	0/0/59/0/0	-	-
		Florideophyceae	Ceramiales	Ceramiaceae	*Ceramium secundatum* Lyngbye, 1819	-	-	-
Chromista	Ochrophyta	Phaeophyceae	Fucales	Durvillaeaceae	*Durvillaea sp.*	-	0/0/8/0/0	-
			Ectocarpales	Chordariaceae	*Leathesia marina* (Lyngbye) Decaisne, 1842	-	-	-
			Scytosiphonales	Scytosiphonaceae	*Petalonia fascia* (O.F.Müller) Kuntze, 1898	-	-	-
					Scytosiphon lomentaria (Lyngbye) Link, 1833	-	-	-

							**NMC3**	
**Kingdom**	**Phylum**	**Class**	**Order**	**Family**	**Species**	**HTS B**	**HTS D**	**Morph.**
Animalia	Annelida	Polychaeta		Capitellidae	*Notomastus profondus* (Eisig, 1887)	-	0/18/40/0/330	0/3/1/2/0
				Maldanidae	*Axiothella constricta* (Claparède, 1869)	0/0/6605/3/0	0/4/7167/0/0	0/1/0/0/0
					Euclymene santandarensis (Rioja, 1917)	0/28132/18991/21806/24830	0/1818/6506/290/9276	0/1/0/0/0
					Euclymene sp.	32146/6/0/8/40	3690/4/5/0/0	-
					Heteroclymene robusta Arwidsson, 1906	0/0/80/100/110	0/1816/6506/290/9276	0/1/0/0/0
					Leiochone leiopygos (Grube, 1860)	0/46973/22387/2356/8859	0/11373/8075/89/2713	-
					Leiochone sp.	4368/4390/1422/4137/27	7139/4232/3710/667/3	-
					Praxillella praetermissa (Malmgren, 1865)	100674/73141/6996/97097/54348	6795/2639/755/2349/15131	1/1/0/0/0
				Orbiniidae	Orbiniidae ni	-	-	-

		Eunicida	Eunicidae	*Marphysa sanguinea* (Montagu, 1815)	8/10/64700/0/0	0/40/60236/0/0	-
				Marphysa sp.	0/0/4/0/0	0/0/155/0/0	-
			Onuphidae	*Diopatra aciculata* Knox & Cameron, 1971	-	-	-
				Diopatra marocensis Paxton, Fadlaoui & Lechapt, 1995	56908/0/7/9/0	1517/4/0/0/0	-
				Diopatra neapolitana Delle Chiaje, 1841	0/97/0/0/9	0/15/0/0/0	-
		Phyllodocida	Glyceridae	*Glycera alba* (O.F. Müller, 1776)	1133/25/96483/27310/10968	1026/152/131646/20396/21114	-
				Glycera sp.	1194/32/103155/28837/11832	1026/152/131585/20389/21105	-
				Glycera tridactyla Schmarda, 1861	-	0/0/88/9/19	-
			Nephtyidae	*Nephtys cirrosa* Ehlers, 1868	0/0/0/0/16	-	-
				Nephtys hombergii Savigny in Lamarck, 1818	0/0/13/0/30	0/0/3/0/24	-
			Nereididae	*Hediste diversicolor* (O.F. Müller, 1776)	-	-	-
			Phyllodocidae	*Phyllodoce groenlandica* Örsted, 1842	-	-	-
			Polynoidae	*Paralepidonotus ampulliferus* (Grube, 1878)	-	-	-
		Sabellida	Oweniidae	*Owenia fusiformis* Delle Chiaje, 1844	-	-	-
		Terebellida	Cirratulidae	Cirratulidae ni	-	-	-
			Pectinariidae	*Lagis koreni* Malmgren, 1866	-	-	-
			Terebellidae	*Pista cristata* (Müller, 1776)	19917/0/187/12/69	34751/93/211/8/4	-
			Trichobranchinae	*Trichobranchus glacialis* Malmgren, 1866	0/0/0/0/8	0/17/0/0/4	-
	Clitellata	Haplotaxida	Tubificidae	*Chaetogaster diaphanus* (Gruithuisen, 1828)	-	-	-
				Nais communis Piguet, 1906	-	-	-
				Stylaria lacustris (Linnaeus, 1767)	-	-	-
		Lumbriculida	Lumbriculidae	*Lumbriculus variegatus* (Müller, 1774)	-	-	-
Arthropoda	Cirripedia	Sessilia	Balanidae	*Amphibalanus amphitrite* (Darwin, 1854)	-	4/0/0/0/0	-
	Copepoda	Calanoida	Acartiidae	*Paracartia grani* Sars G.O., 1904	-	-	-
			Centropagidae	*Centropages hamatus* (Lilljeborg, 1853)	-	-	-
			Temoridae	*Temora longicornis* (Müller O.F., 1785)	-	-	-
		Cyclopoida		Cyclopoida ni	-	3/0/0/0/0	-
		Poecilostomatoida	Mytilicolidae	*Mytilicola orientalis* Mori, 1935	-	-	-
	Malacostraca	Amphipoda	Ampeliscidae	*Ampelisca brevicornis* (Costa, 1853)	-	0/0/14/0/0	1/0/0/0/1
				Ampelisca sp.	0/372/115/629/0	0/12/307/7/0	-
			Corophiidae	*Corophium sp.*	0/0/183/16/173	0/0/80/4/34	-
		Isopoda	Anthuridae	*Cyathura carinata* (Krøyer, 1847)	-	-	0/0/0/1/0
			Sphaeromatidae	Sphaeromatidae ni	-	-	-
		Decapoda	Callianassidae	*Pestarella tyrrhena* (Petagna, 1792)	-	0/3/0/0/0	-

				Diogenidae	*Diogenes pugilator* (Roux, 1829)	0/0/0/1194/0	0/0/0/89/0	-
				Inachidae	*Macropodia rostrata* (Linnaeus, 1761)	13/0/0/0/0	-	-
					Macropodia sp.	838/0/13/0/0	24/0/6/0/0	-
				Paguridae	Pylopaguropsis magnimanus	0/0/0/23406/0	0/16/0/26927/0	-
				Pilumnidae	*Pilumnus hirtellus* (Linnaeus, 1761)	-	-	-
					Pilumnus spinifer H. Milne Edwards, 1834	-	-	-
				Pinnotheridae	*Pinnotheres pisum* (Linnaeus, 1767)	0/0/0/0/23	0/0/0/0/3	-
				Upogebiidae	*Upogebia deltaura* (Leach, 1815)	-	-	-
	Bryozoa	Gymnolaemata	Cheilostomatida	Bugulidae	*Bugula neritina* (Linnaeus, 1758)	-	-	-
	Echinodermata	Echinoidea	Spatangoida	Loveniidae	*Echinocardium cordatum* (Pennant, 1777)	0/0/0/35176/5	-	-
	Mollusca	Bivalvia	Anomalodesmata	Thraciidae	*Thracia phaseolina* (Lamarck, 1818)	6/0/0/0/0	17/0/0/0/0	-
			Myoida	Corbulidae	*Corbula gibba* (Olivi, 1792)	-	-	-
			Veneroida	Cardiidae	*Cerastoderma edule* (Linnaeus, 1758)	-	-	-
					Parvicardium exiguum (Gmelin, 1791)	-	-	-
					Parvicardium pinnulatum (Conrad, 1831)	-	-	-
				Donacidae	*Donax trunculus* Linnaeus, 1758	-	-	2/0/0/0/0
				Mactridae	*Spisula solida* (Linnaeus, 1758)	-	-	-
				Semelidae	*Abra alba* (W. Wood, 1802)	-	-	0/0/0/0/1
				Ungulinidae	*Diplodonta rotundata* (Montagu, 1803)	-	-	-
				Veneridae	*Chamelea striatula* (da Costa, 1778)	8/0/0/0/0	112/0/0/0/0	-
			[unassigned] Euheterodonta	Solenidae	*Solen marginatus* Pulteney, 1799	-	-	-
		Gastropoda	Littorinimorpha	Hydrobiidae	*Ecrobia ventrosa* (Montagu, 1803)	-	-	-
			Neogastropoda	Nassariidae	*Nassarius reticulatus* (Linnaeus, 1758)	0/12/0/3/0	4/3/0/5/0	-
	Nemertea	Anopla		Lineidae	*Cerebratulus longiceps* Coe, 1901	-	-	-
					Cerebratulus sp.	11/0/0/53/0	4/0/0/0/0	-
Plantae	Rhodophyta	Bangiophyceae	Bangiales	Bangiaceae	*Pyropia haitanensis* (T.J.Chang & B.F.Zheng) N.Kikuchi & M.Miyata, 2011	-	-	-
		Florideophyceae	Ceramiales	Ceramiaceae	*Ceramium secundatum* Lyngbye, 1819	-	-	-
Chromista	Ochrophyta	Phaeophyceae	Fucales	Durvillaeaceae	*Durvillaea sp.*	-	-	-
			Ectocarpales	Chordariaceae	*Leathesia marina* (Lyngbye) Decaisne, 1842	-	0/0/0/13/0	-
			Scytosiphonales	Scytosiphonaceae	*Petalonia fascia* (O.F.Müller) Kuntze, 1898	0/0/0/44/0	0/0/0/7/0	-
					Scytosiphon lomentaria (Lyngbye) Link, 1833	0/0/0/8/0	0/0/0/4/0	-

NMC4

Kingdom	Phylum	Class	Order	Family	Species	HTS B	HTS D	Morph.
Animalia	Annelida	Polychaeta		Capitellidae	*Notomastus profondus* (Eisig, 1887)	-	0/0/0/0/39	2/2/1/1/2
				Maldanidae	*Axiothella constricta* (Claparède, 1869)	-	-	0/1/0/0/2
					Euclymene santandarensis (Rioja, 1917)	6/0/14/0/94	0/4/0/0/0	-
					Euclymene sp.	81884/0/3/4/17379	4141/0/0/0400	-
					Heteroclymene robusta Arwidsson, 1906	0/8/54/5/0	-	-
					Leiochone leiopygos (Grube, 1860)	66883/0/3682/0/5683	23424/4/93/13/47	-
					Leiochone sp.	5481/8529/19580/0/5	6578/7055/9972/9/0	-
					Praxillella praetermissa (Malmgren, 1865)	17/10096/9/214979/143	16/84/3/33374/0	-
				Orbiniidae	Orbiniidae ni	0/0/0/0/110	-	-
			Eunicida	Eunicidae	*Marphysa sanguinea* (Montagu, 1815)	14/0/0/0/0	0/3/0/0/0	-
					Marphysa sp.	-	-	-
				Onuphidae	*Diopatra aciculata* Knox & Cameron, 1971	-	-	-
					Diopatra marocensis Paxton, Fadlaoui & Lechapt, 1995	0/0/0/7/0	0/0/0/4/0	-
					Diopatra neapolitana Delle Chiaje, 1841	3/0/0/0/100	0/36/0/0/0	-
			Phyllodocida	Glyceridae	*Glycera alba* (O.F. Müller, 1776)	2824/26489/96088/5/18	3083/37774/68109/6/0	-
					Glycera sp.	3017/27374101978/7/21	3083/37768/68083/6/0	-
					Glycera tridactyla Schmarda, 1861	-	0/15/44/0/0	-
				Nephtyidae	*Nephtys cirrosa* Ehlers, 1868	-	-	-
					Nephtys hombergii Savigny in Lamarck, 1818	6/0/43/3/7	0/0/5/0/0	-
				Nereididae	*Hediste diversicolor* (O.F. Müller, 1776)	-	-	-
				Phyllodocidae	*Phyllodoce groenlandica* Örsted, 1842	-	-	-
				Polynoidae	*Paralepidonotus ampulliferus* (Grube, 1878)	-	-	-
			Sabellida	Oweniidae	*Owenia fusiformis* Delle Chiaje, 1844	-	-	-
			Terebellida	Cirratulidae	Cirratulidae ni	-	-	-
				Pectinariidae	*Lagis koreni* Malmgren, 1866	219/0/0/0/0	984/0/0/0/0	-
				Terebellidae	*Pista cristata* (Müller, 1776)	15462/97719/14039/7208/37	29861/88881/11545/12673/3	0/1/0/1/0
				Trichobranchinae	*Trichobranchus glacialis* Malmgren, 1866	3038/0/0/3/0	17917/0/6/3/0	-
		Clitellata	Haplotaxida	Tubificidae	*Chaetogaster diaphanus* (Gruithuisen, 1828)	-	-	-
					Nais communis Piguet, 1906	-	-	-
					Stylaria lacustris (Linnaeus, 1767)	0/0/6/0/4	0/4/0/0/44	-
			Lumbriculida	Lumbriculidae	*Lumbriculus variegatus* (Müller, 1774)	9/0/0/0/0	-	-
	Arthropoda	Cirripedia	Sessilia	Balanidae	*Amphibalanus amphitrite* (Darwin, 1854)	-	-	-
		Copepoda	Calanoida	Acartiidae	*Paracartia grani* Sars G.O., 1904	-	-	-

			Centropagidae	*Centropages hamatus* (Lilljeborg, 1853)	-	-	-
			Temoridae	*Temora longicornis* (Müller O.F., 1785)	0/0/0/10/0	0/0/0/25/0	-
		Cyclopoida		Cyclopoida ni	-	-	-
		Poecilostomatoida	Mytilicolidae	*Mytilicola orientalis* Mori, 1935	-	0/0/39/0/0	-
	Malacostraca	Amphipoda	Ampeliscidae	*Ampelisca brevicornis* (Costa, 1853)	-	0/0/0/20/0	-
				Ampelisca sp.	0/0/0/1693/0	0/0/0/720/0	-
			Corophiidae	*Corophium sp.*	0/8/0/0/0	-	-
		Isopoda	Anthuridae	*Cyathura carinata* (Krøyer, 1847)	-	-	1/0/1/0/1
			Sphaeromatidae	Sphaeromatidae ni	0/0/0/0/548		
		Decapoda	Callianassidae	*Pestarella tyrrhena* (Petagna, 1792)	2206/0/0/0/0	1152/0/0/0/0	-
			Diogenidae	*Diogenes pugilator* (Roux, 1829)	-	-	-
			Inachidae	*Macropodia rostrata* (Linnaeus, 1761)	-	-	-
				Macropodia sp.	-	-	-
			Paguridae	Pylopaguropsis magnimanus	-	-	-
			Pilumnidae	*Pilumnus hirtellus* (Linnaeus, 1761)	0/0/6/14/0	-	-
				Pilumnus spinifer H. Milne Edwards, 1834	-	-	-
			Pinnotheridae	*Pinnotheres pisum* (Linnaeus, 1767)	0/15402/12676/0/845	0/716/479/0/16	0/0/0/1/0
			Upogebiidae	*Upogebia deltaura* (Leach, 1815)	-	-	-
Bryozoa	Gymnolaemata	Cheilostomatida	Bugulidae	*Bugula neritina* (Linnaeus, 1758)	0/0/51/0/0	0/0/0/12/0	-
Echinodermata	Echinoidea	Spatangoida	Loveniidae	*Echinocardium cordatum* (Pennant, 1777)	4/0/0/0/0	-	0/0/0/0/1
Mollusca	Bivalvia	Anomalodesmata	Thraciidae	*Thracia phaseolina* (Lamarck, 1818)	0/0/0/0/117	0/0/0/0/135	-
		Myoida	Corbulidae	*Corbula gibba* (Olivi, 1792)	-	-	0/1/0/1/0
		Veneroida	Cardiidae	*Cerastoderma edule* (Linnaeus, 1758)	0/0/22/0/21	0/5/129/0/105	0/2/1/1/1
				Parvicardium exiguum (Gmelin, 1791)	-	-	-
				Parvicardium pinnulatum (Conrad, 1831)	-	-	-
			Donacidae	*Donax trunculus* Linnaeus, 1758	-	-	-
			Mactridae	*Spisula solida* (Linnaeus, 1758)	-	-	0/0/0/0/1
			Semelidae	*Abra alba* (W. Wood, 1802)	-	-	0/0/0/1/0
			Ungulinidae	*Diplodonta rotundata* (Montagu, 1803)	-	-	-
			Veneridae	*Chamelea striatula* (da Costa, 1778)	-	-	-
		[unassigned] Euheterodonta	Solenidae	*Solen marginatus* Pulteney, 1799	-	-	0/0/1/1/0
	Gastropoda	Littorinimorpha	Hydrobiidae	*Ecrobia ventrosa* (Montagu, 1803)	-	-	-
		Neogastropoda	Nassariidae	*Nassarius reticulatus* (Linnaeus, 1758)	0/4/0/0/12	0/16/0/0/0	-
Nemertea	Anopla		Lineidae	*Cerebratulus longiceps* Coe, 1901	-	0/0/0/4/0	-

					Cerebratulus sp.	-	-	-
Plantae	Rhodophyta	Bangiophyceae	Bangiales	Bangiaceae	*Pyropia haitanensis* (T.J.Chang & B.F.Zheng) N.Kikuchi & M.Miyata, 2011	0/0/0/3/0	-	-
		Florideophyceae	Ceramiales	Ceramiaceae	*Ceramium secundatum* Lyngbye, 1819	0/0/104/0/0	0/0/0/272/0	-
Chromista	Ochrophyta	Phaeophyceae	Fucales	Durvillaeaceae	*Durvillaea sp.*	-	-	-
			Ectocarpales	Chordariaceae	*Leathesia marina* (Lyngbye) Decaisne, 1842	-	-	-
			Scytosiphonales	Scytosiphonaceae	*Petalonia fascia* (O.F.Müller) Kuntze, 1898	-	-	-
					Scytosiphon lomentaria (Lyngbye) Link, 1833	-	-	-
